# Supplementary material for: ezSingleCell: an integrated one-stop single-cell and spatial omics analysis platform for bench scientists
Source: Nat Commun. 2024 Jul 3;15:5600. doi: 10.1038/s41467-024-48188-2 (PMC11222513; doi:10.1038/s41467-024-48188-2)
Supplement: Supplementary file 1 — Supplementary Information [file 41467_2024_48188_MOESM1_ESM.pdf]

Supplementary Materials for

ezSingleCell: an integrated one-stop single-cell and spatial omics analysis platform for bench scientists

Raman Sethi[1], Kok Siong Ang[2], Mengwei Li[2], Yahui Long[2], Jingjing Ling[4], Jinmiao Chen[1,2,3*]

[1] Bioinformatics Institute (BII), Agency for Science, Technology and Research (A*STAR), 30 Biopolis St, Matrix, Singapore 138671
[2] Institute of Molecular and Cell Biology (IMCB), Agency for Science, Technology and Research (A*STAR), 61 Biopolis Dr, Proteos, Singapore 138673
[3] Immunology Translational Research Program, Department of Microbiology and Immunology, Yong Loo Lin School of Medicine, National University of Singapore (NUS), 5 Science Drive 2, Blk MD4, Level 3, Singapore 117545
[4] Singapore Immunology Network (SIgN), Agency for Science, Technology and Research (A*STAR), 8A Biomedical Grove, Immunos, Singapore 138648, Singapore

[*]Corresponding author. Email: chen_jinmiao@bii.a-star.edu.sg

This Supplementary Information file contains:

Supplementary Figures 1 to 5
Supplementary Tables 1 to 3

Supplementary Figures

Figure Suppl. S1. Web interface of the ezSingleCell webserver

Figure Suppl. S2. Overview of ezSingleCell’s five modules

Figure Suppl. S3. ezSingleCell scRNA-seq workflow

Figure Suppl. S4. ezSingleCell scIntegration workflow

Figure Suppl. S5. ezSingleCell spatial transcriptomics workflow

Supplementary Tables

Table Suppl. S1. List of publicly available single-cell web servers

Table Suppl. S2. List of tools used in the five modules of ezSingleCell along their web addresses

Table Suppl. S3. List of datasets used in ezSingleCell as example datasets for each module

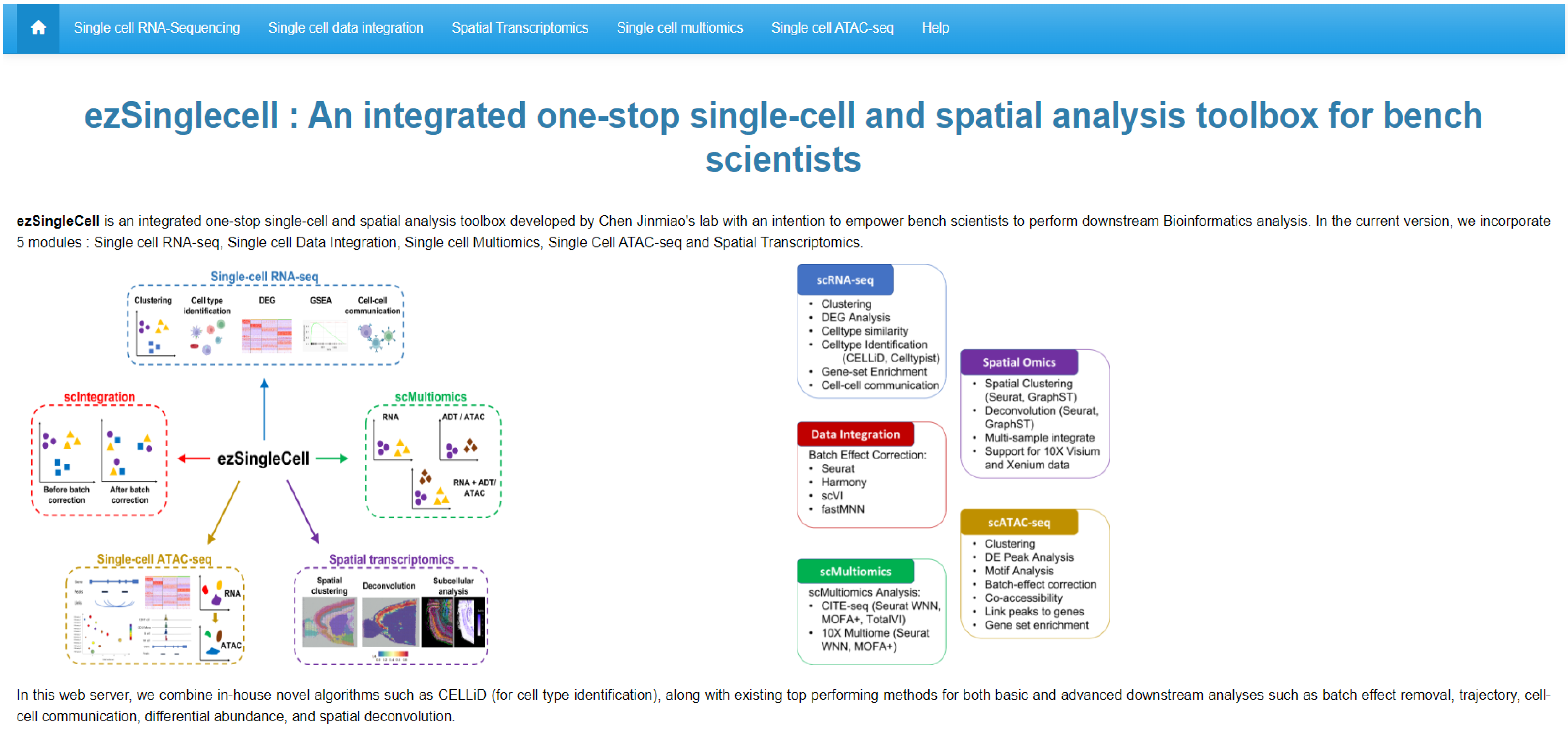

Suppl Fig S1. Web interface of the ezSingleCell webserver. Users can perform data analysis with five different modules, scRNA-seq, scIntegration, scMultiomics, scATAC-seq, and Spatial Transcriptomics. The web server is hosted at https://immunesinglecell.org/ezsc/.

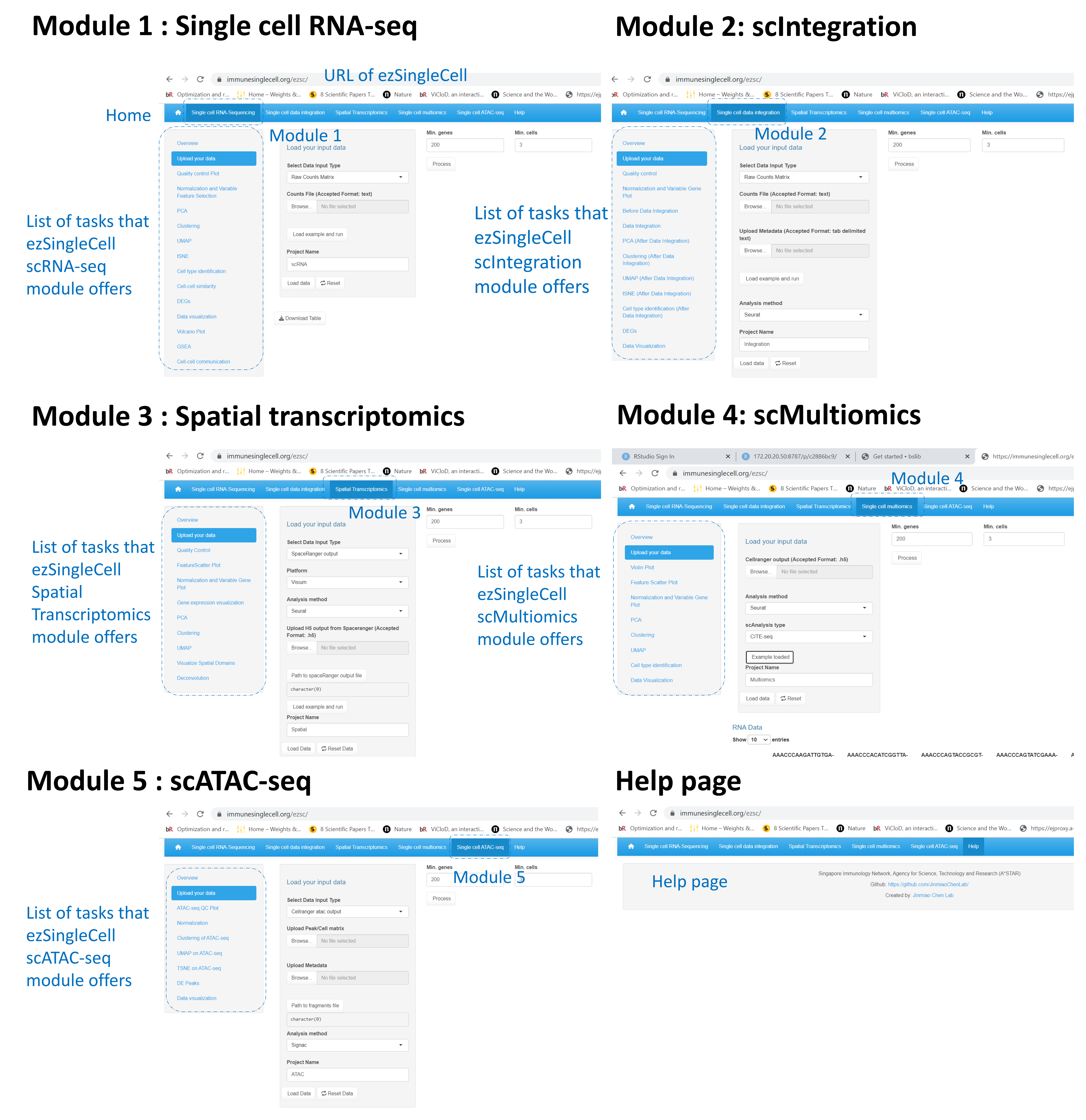

Suppl Fig S2. Overview of ezSingleCell's five modules (scRNA-seq, scIntegration, scMultiomics, scATAC-seq, and Spatial Transcriptomics) along with the help page containing in-depth manuals for running analysis tasks in each module.

A. Quality control plots

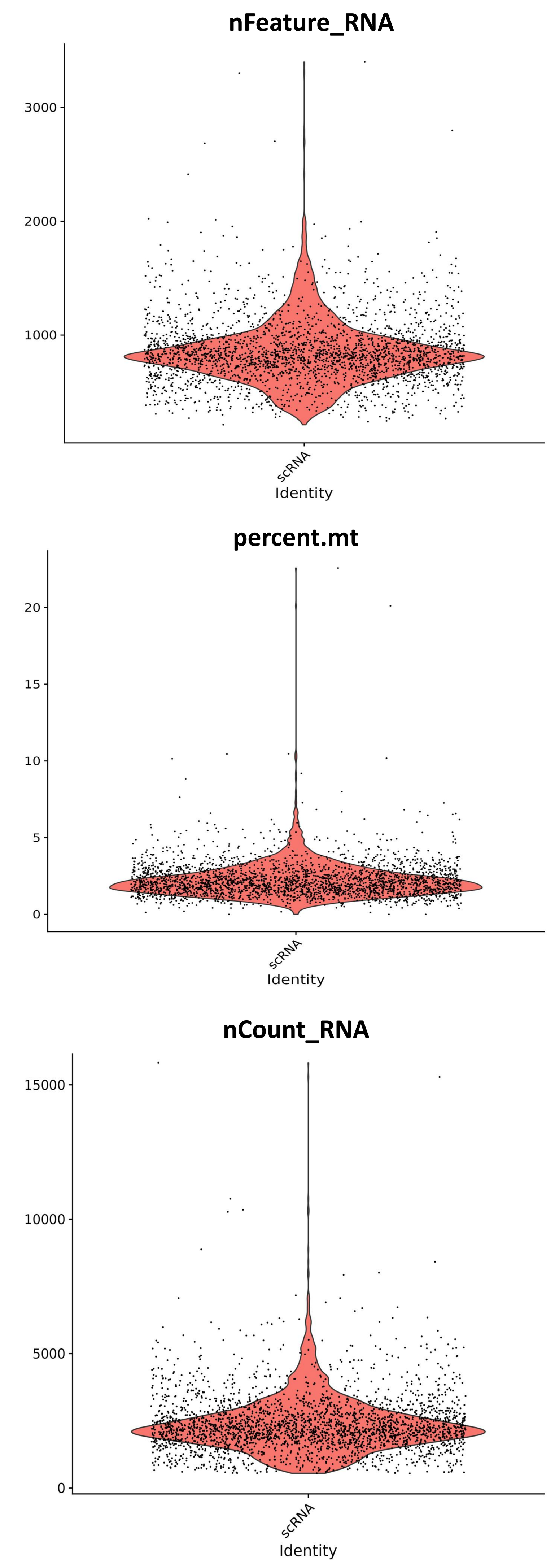

B. Cell type identification using CELLiD

Cluster	primary.predict	Ground Truth
0	Memory CD4 T cell	Memory CD4 T
1	Naive CD4 T cell	Naïve CD4 T
2	GZMB CD8 T cell	CD8 T
3	CD14 monocyte	CD14+ Mono
4	Memory B cell	B cells
5	Naive B cell	B cells
6	CD16 NK cell	NK
7	CD14 monocyte	CD14+ Mono
8	Memory CD4 T cell	Memory CD4 T
9	CD16 monocyte	FCGR3A+ Mono
10	Naive CD4 T cell	Naïve CD4 T
11	GZMK CD8 T cell	CD8 T
12	CD14 monocyte	CD14+ Mono
13	Dendritic cell	DC
14	Megakaryocyte	Platelet

C. Violin plot

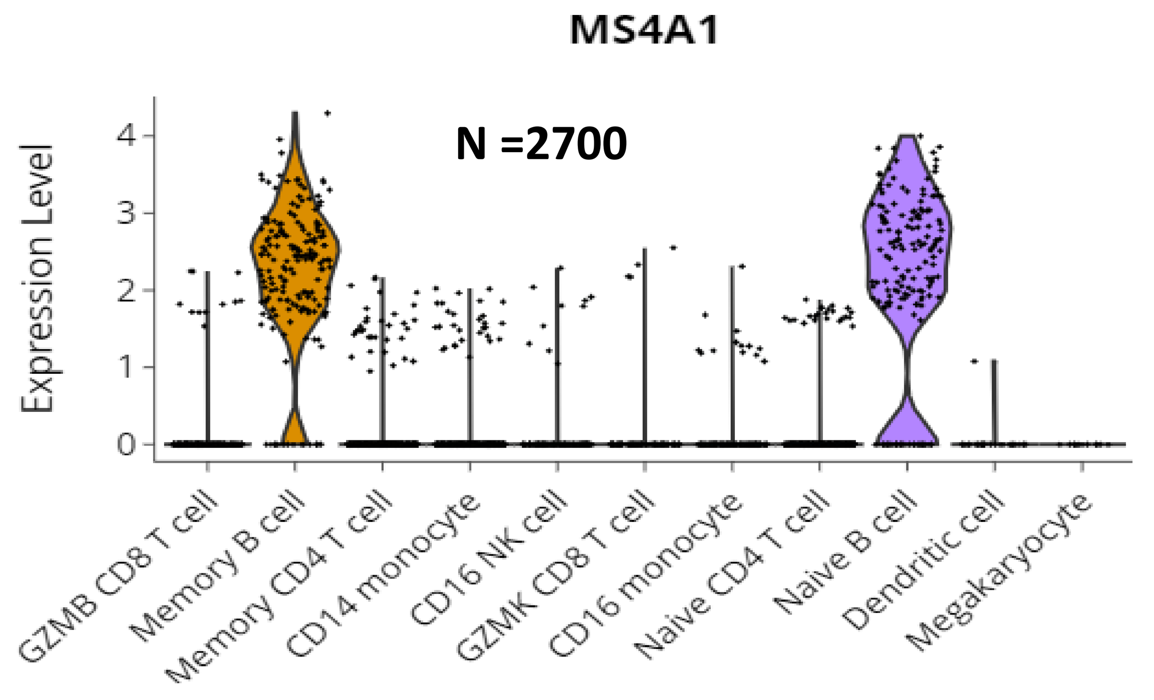

D. Feature plot

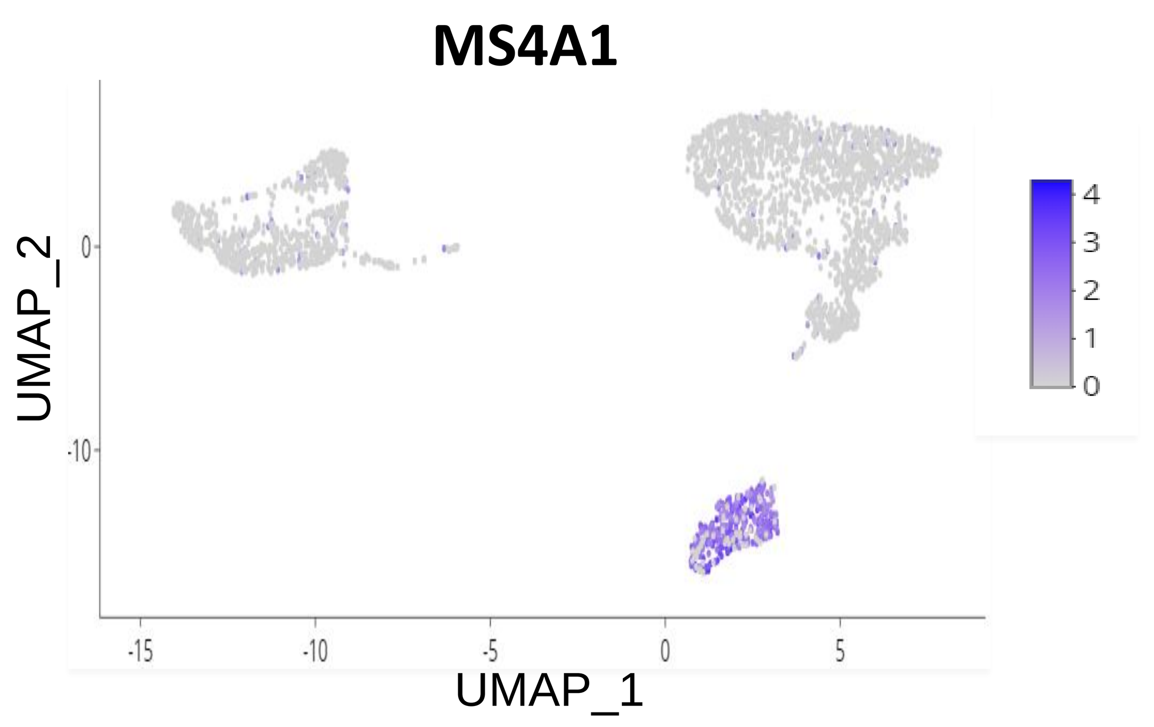

E. Ridge plot

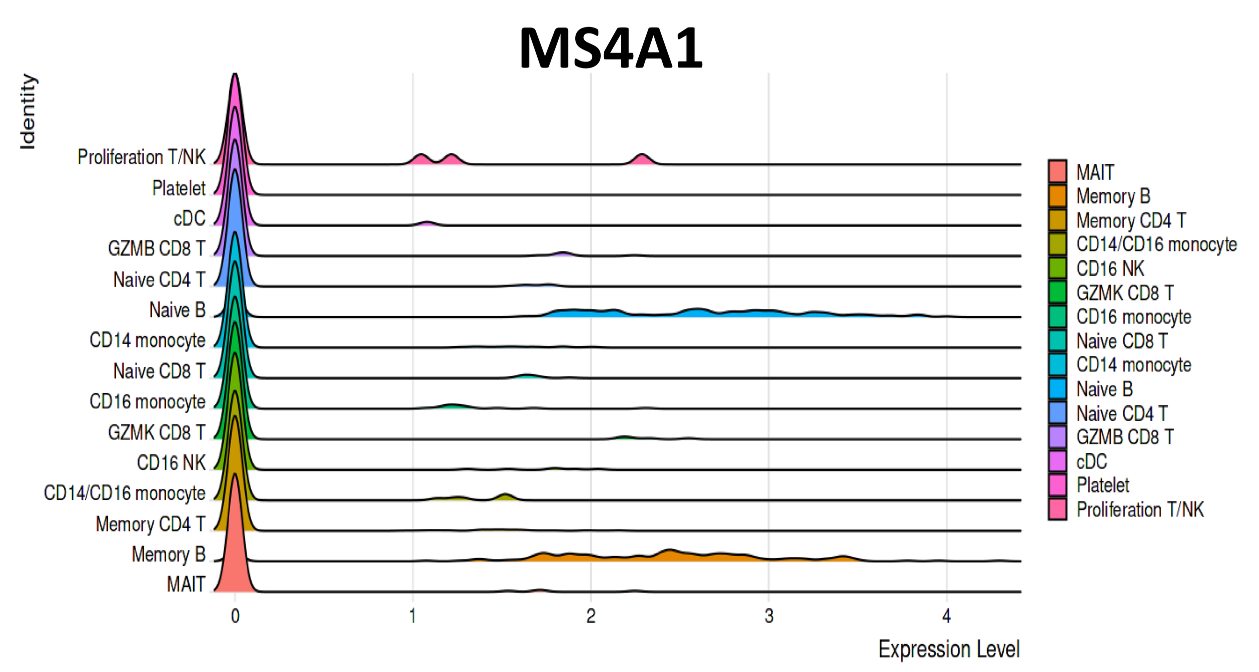

F. Violin plot of memory B cells

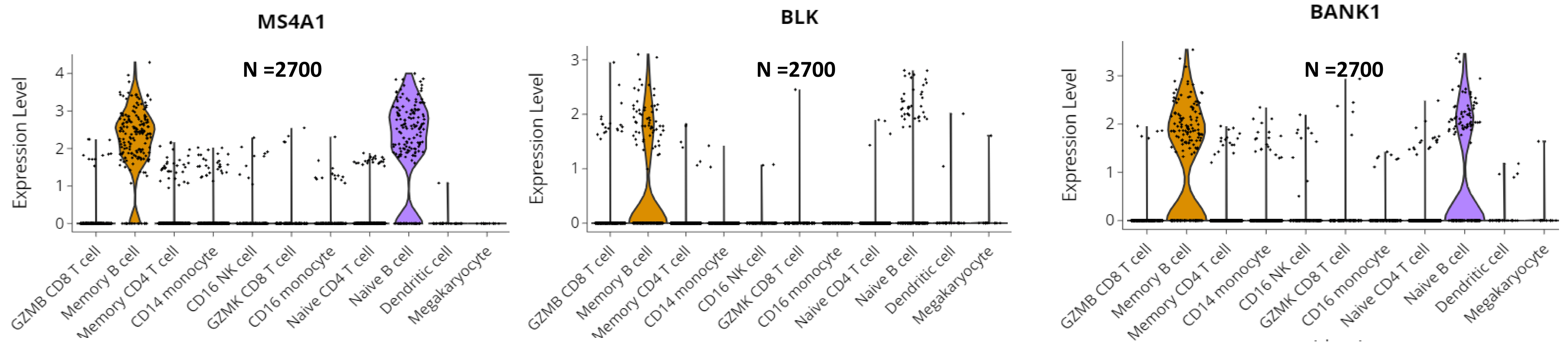

G. Violin plot of naive B cells

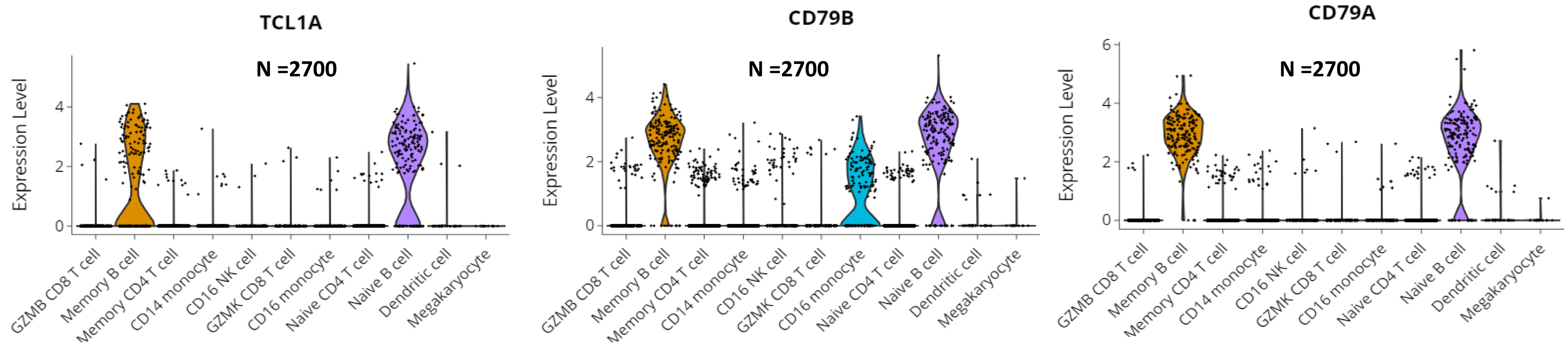

Supp Fig S3. ezSingleCell scRNA-seq workflow. (A) Quality control; (B-E) Comparison of cell types identified using CELLiD with the ground truth annotation and marker genes visualization using violin plots, feature plots, and ridgeline plot; (F-G) Violin plot of marker genes for naïve and memory B cells.

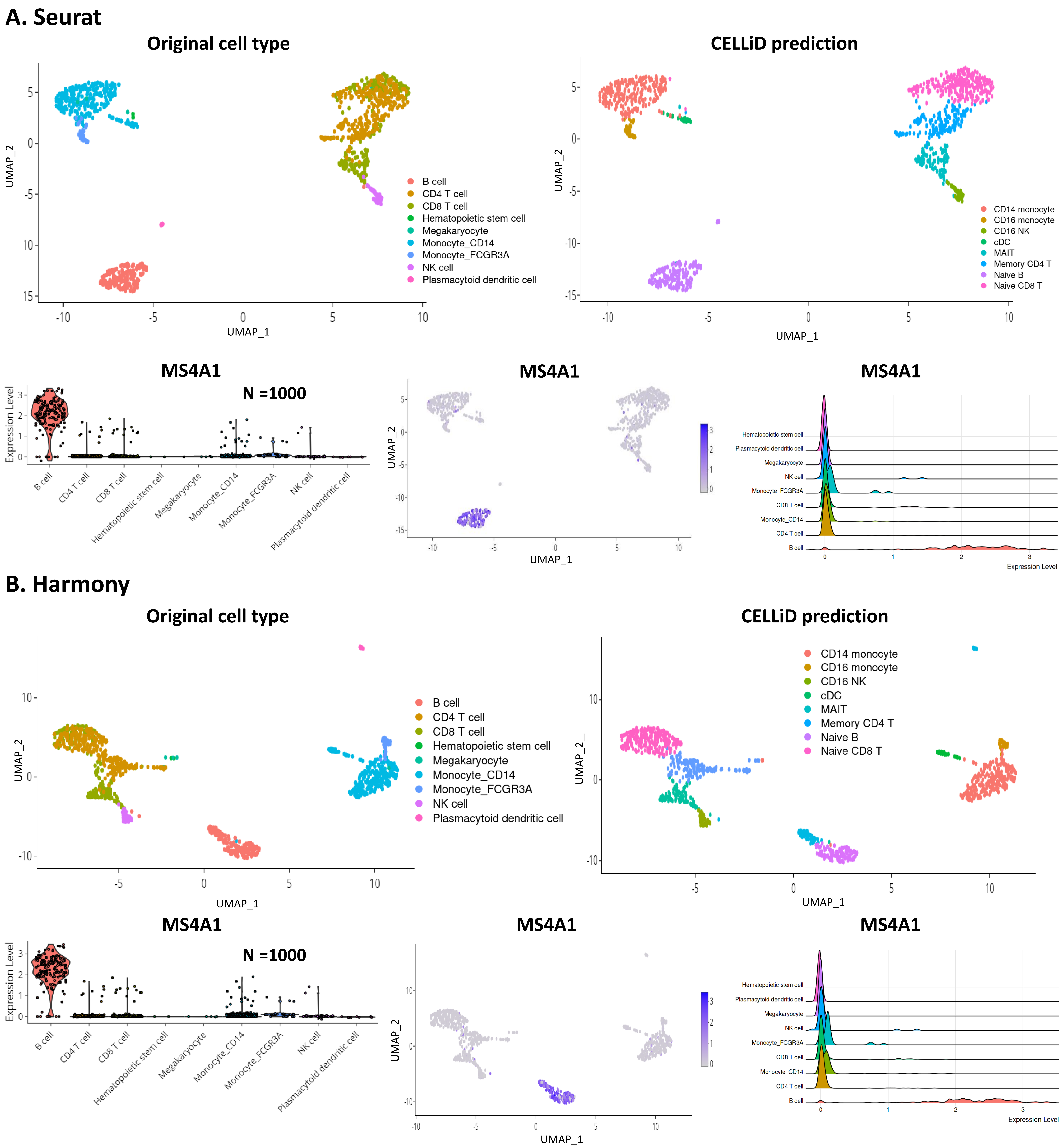

Supp Fig S4. ezSingleCell scIntegration workflow. The workflow provides cell type identification and data visualization (violin plot, feature plot, and ridgeline plot) using batch corrected values from methods available on the ezSingleCell server such as (A) Seurat and (B) Harmony.

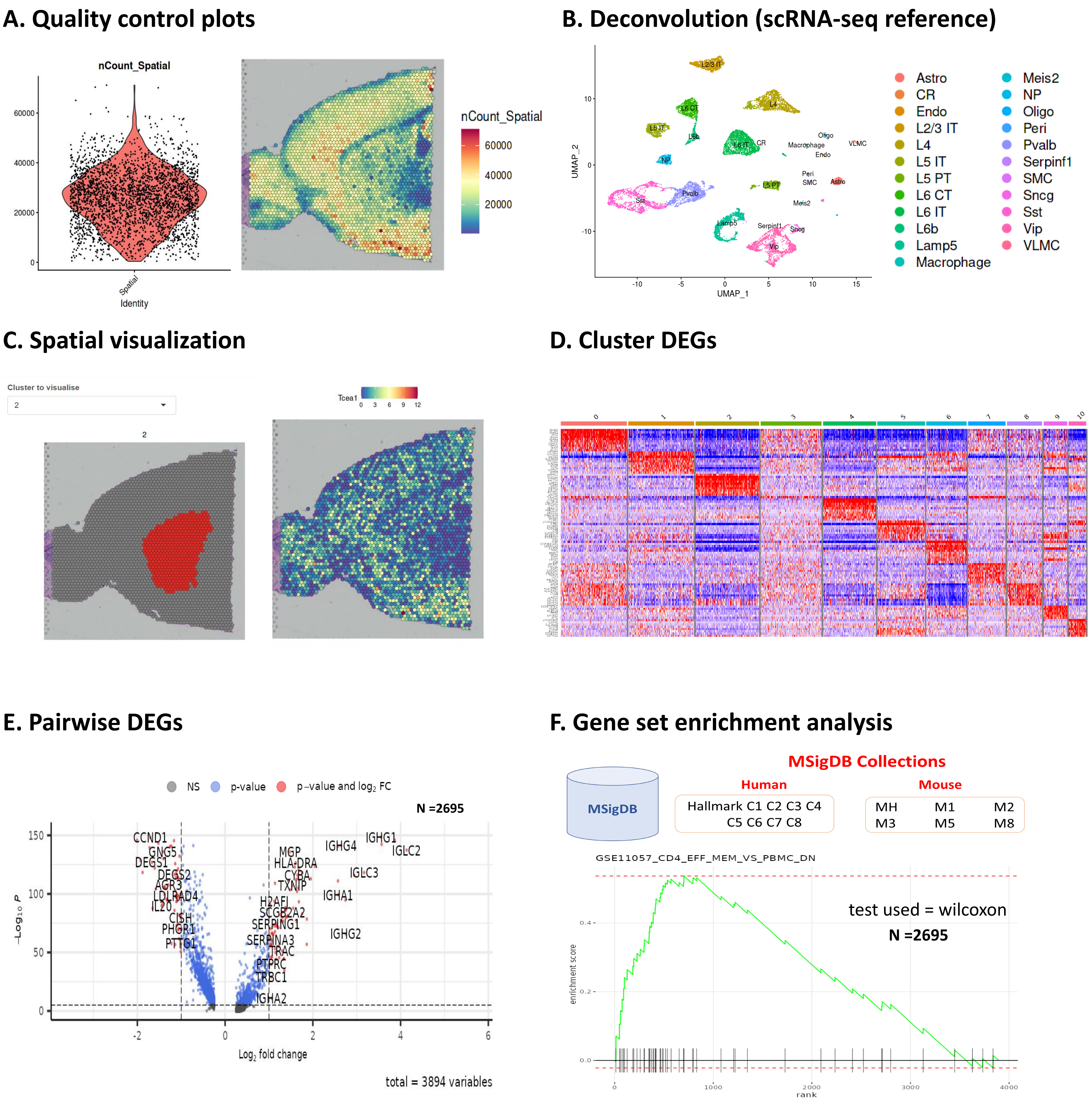

Supp Fig S5. ezSingleCell spatial transcriptomics workflow. (A) Quality control; (B) Visualization of scRNA-seq reference data for deconvolution; (C) Spatial visualization (spatial domain and gene expression); (D) Cluster DEGs; (E) Pairwise DEGs; (F) Gene set enrichment analysis of spatial data using fgsea. We used the weighted Kolmogorov–Smirnov statistic for GSEA analysis.

Webserver	Link	Type	Reference
ICARUS	https://launch.icarus-scrnaseq.cloud.edu.au/	Academic	Jiang A et al., 2022
Cellar	https://cellar.cmu.hubmapconsortium.org/app/cellar	Academic	Hasanaj E et al., 2022
ASAP	https://asap.epfl.ch/	Academic	Gardeux V et al., 2017
ASAP2020	https://asap.epfl.ch/	Academic	David FPA, 2020
alona	https://alona.panglaodb.se/	Academic	Franzén O et al., 2020
SCiAp	https://humancellatlas.usegalaxy.eu/	Academic	Moreno P et al., 2021
NASQAR	https://nasqar.abudhabi.nyu.edu/SeuratV3Wizard/	Academic	Yousif A et al., 2020
SCTK	https://www.camplab.net/sctk/	Academic	Hong R et al., 2022
SCTK2.0	https://www.camplab.net/sctk/	Academic	Wang Y et al., 2022
Asc-Seurat	https://asc-seurat.readthedocs.io/en/latest/index.html	Academic	Pereira WJ et al., 2021
shinyGO	http://bioinformatics.sdstate.edu/go/	Academic	Ge SX et al., 2020
ClusterProfShinyGSEA	https://nasqar.abudhabi.nyu.edu/ClusterProfShinyGSEA/	Academic	Yousif A et al., 2020
ShinyArchR.Uio	https://cancell.medisin.uio.no/ShinyArchR.UiO/	Academic	Sharma A et al., 2022
10X Loupe Browser	https://www.10xgenomics.com/products/loupe-browser	Commercial	-
Partek	https://www.partek.com/partek-flow/	Commercial	-
Bioturing	https://bioturing.com/bbrowser	Commercial	-

Suppl. Table 1. List of publicly available single cell web servers (both academic and commercial) along with their web addresses.

Module	Tool name	Description	Link	Reference
scRNA-seq	Seurat	Single cell clustering and Dimension Reduction	https://satijalab.org/seurat/articles/pbmc3k_tutorial.html	Hao Y et al., 2021
	CELLiD	Cell type identification	https://www.immunesinglecell.org/cellpredictor	Li M et al., 2021
	CellTypist	Cell type identification	https://www.celltypist.org/	Chuan X et al., 2023; Conde CD et al., 2022
	fgsea	Gene set enrichment analysis (GSEA)	https://www.gsea-msigdb.org/gsea/index.jsp	Subramanian A et al., 2007
	CellphoneDB	Cell-cell communication	https://www.cellphonedb.org/	Efremova M et al., 2020
scIntegration	Seurat	Single-cell data integration	https://satijalab.org/seurat/articles/integration_introduction.html	Hao Y et al., 2021
	Harmony	Single-cell data integration	https://github.com/immunogenomics/harmony	Korsunsky I et al., 2019
	scVI	Single-cell data integration	https://github.com/scverse/scvi-tools	Lopez R et al., 2018
	fastMNN	Single-cell data integration	https://rdrr.io/github/LTLA/batchelor/man/fastMNN.html	Haghverdi L et al., 2018
	fgsea	Gene set enrichment analysis (GSEA)	https://www.gsea-msigdb.org/gsea/index.jsp	Subramanian A et al., 2007
	CellphoneDB	Cell-cell communication	https://www.cellphonedb.org/	Efremova M et al., 2020
	CELLiD	Cell type identification	https://www.immunesinglecell.org/cellpredictor	Li M et al., 2021
	CellTypist	Cell type identification	https://www.celltypist.org/	Chuan X et al., 2023; Conde CD et al., 2022
scMultiomics	Seurat	Single cell multiomics analysis	https://satijalab.org/seurat/articles/multimodal_vignette.html	Hao Y et al., 2021
	MOFA+	Single cell multiomics analysis	https://biofam.github.io/MOFA2/	Argelaguet R et al., 2020
	fgsea	Gene set enrichment analysis (GSEA)	https://www.gsea-msigdb.org/gsea/index.jsp	Subramanian A et al., 2007
	CELLiD	Cell type identification	https://www.immunesinglecell.org/cellpredictor	Li M et al., 2021
	CellTypist	Cell type identification	https://www.celltypist.org/	Chuan X et al., 2023; Conde CD et al., 2022
scATAC-seq	Signac	Single-cell ATAC-seq analysis	https://stuartlab.org/signac/articles/overview.html	Stuart T et al., 2021
	fgsea	Gene set enrichment analysis (GSEA)	https://www.gsea-msigdb.org/gsea/index.jsp	Subramanian A et al., 2007
	rGREAT	Gene set enrichment analysis (GSEA)	https://github.com/jokergoo/rGREAT	Gu Z et al., 2022
Spatial Transcriptomics	Seurat	Spatial clustering and deconvolution	https://satijalab.org/seurat/articles/spatial_vignette.html	Hao Y et al., 2021
	GraphST	Spatial clustering and deconvolution	https://github.com/JinmiaoChenLab/DeepST	Long Y et al., 2022
	fgsea	Gene set enrichment analysis (GSEA)	https://www.gsea-msigdb.org/gsea/index.jsp	Subramanian A et al., 2007

Suppl Table 2. List of packages used in the five modules of ezSingleCell (scRNA-seq, scIntegration, scMultiomics, scATAC-seq, and Spatial Transcriptomics) along with the detailed functionalities of each package and their web addresses.

Module	Dataset description	Data Available from	Total number of cells/spots	Technology used
scRNA-Seq	2,700 Human peripheral blood mononuclear cells (Seurat guided clustering tutorial dataset)	10x genomics https://cf.10xgenomics.com/samples/cell/pbmc3k/pbmc3k filtered gene bc matrices.tar.gz	2700	10X
scIntegration	Human PBMC (Batch 1)	https://support.10xgenomics.com/single-cell-gene-expression/datasets/2.1.0/pbmc8k	500	10X 3'
	Human PBMC (Batch 2)	https://support.10xgenomics.com/single-cell-vdj/datasets/2.2.0/vdj_v1_hs_pbmc_5gex	500	10X 5'
scMultiomics	CITE-Seq: peripheral blood mononuclear cells (PBMCs) from a healthy donor	https://support.10xgenomics.com/single-cell-gene-expression/datasets/3.0.0/pbmc_10k_protein_v3	7865	10X
	10X MultiOme: peripheral blood mononuclear cells (PBMCs) from a healthy donor	https://support.10xgenomics.com/single-cell-multiome-atac-gex/datasets/1.0.0/pbmc_unsorted_3k?	3012	10X
scATAC-seq	500 peripheral blood mononuclear cells from a healthy donor	https://www.10xgenomics.com/resources/datasets/500-peripheral-blood-mononuclear-cells-pbm-cs-from-a-healthy-donor-next-gem-v-1-1-1-1-standard-2-0-0	500	10X
	10k peripheral blood mononuclear cells from a healthy donor	https://www.10xgenomics.com/datasets/10-k-peripheral-blood-mononuclear-cells-pbm-cs-from-a-healthy-donor-next-gem-v-1-1-1-1-standard-2-0-0	10000	10X
	Batch1 scATAC-seq: 500 peripheral blood mononuclear cells (PBMCs) from a healthy donor	https://www.10xgenomics.com/resources/datasets/500-peripheral-blood-mononuclear-cells-pbm-cs-from-a-healthy-donor-v-1-0-1-1-standard-1-1-0	500	10X
	Batch2 scATAC-seq: 1k peripheral blood mononuclear cells (PBMCs) from a healthy donor	https://www.10xgenomics.com/resources/datasets/1-k-peripheral-blood-mononuclear-cells-pbm-cs-from-a-healthy-donor-v-1-0-1-1-standard-1-1-0	1000	10X
Spatial Transcriptomics	Mouse brain sagittal anterior	https://www.10xgenomics.com/resources/datasets/mouse-brain-serial-section-1-sagittal-anterior-1-standard-1-1-0	2695	10X
	Human Breast Cancer Block A	https://www.10xgenomics.com/resources/datasets/human-breast-cancer-block-a-section-1-1-standard-1-1-0	3798	10X
	Fresh frozen mouse brain (Xenium)	https://www.10xgenomics.com/resources/datasets/fresh-frozen-mouse-brain-for-xenium-explorer-demo-1-standard	36,602 molecules	10X

Suppl. Table 3. List of datasets used in ezSingleCell as example datasets for each module (scRNA-seq, scIntegration, scMultiomics, scATAC-seq, and Spatial Transcriptomics). These datasets were downloaded from 10X Genomics website (https://www.10xgenomics.com/resources/datasets).
