# Supplementary material for: ezSingleCell: an integrated one-stop single-cell and spatial omics analysis platform for bench scientists
Source: Nat Commun. 2024 Jul 3;15:5600. doi: 10.1038/s41467-024-48188-2 (PMC11222513; doi:10.1038/s41467-024-48188-2)
Supplement: Supplementary file 3 — Reporting Summary [file 41467_2024_48188_MOESM3_ESM.pdf]

nature portfolio

Corresponding author(s): Jinmiao Chen
Last updated by author(s): Mar 14, 2024

Reporting Summary

Nature Portfolio wishes to improve the reproducibility of the work that we publish. This form provides structure for consistency and transparency in reporting. For further information on Nature Portfolio policies, see our Editorial Policies and the Editorial Policy Checklist.

Statistics

For all statistical analyses, confirm that the following items are present in the figure legend, table legend, main text, or Methods section.

n/a	Confirmed	
☐	☒	The exact sample size (*n*) for each experimental group/condition, given as a discrete number and unit of measurement
☐	☒	A statement on whether measurements were taken from distinct samples or whether the same sample was measured repeatedly
☐	☒	The statistical test(s) used AND whether they are one- or two-sided<br>*Only common tests should be described solely by name; describe more complex techniques in the Methods section.*
☐	☒	A description of all covariates tested
☒	☐	A description of any assumptions or corrections, such as tests of normality and adjustment for multiple comparisons
☒	☐	A full description of the statistical parameters including central tendency (e.g. means) or other basic estimates (e.g. regression coefficient) AND variation (e.g. standard deviation) or associated estimates of uncertainty (e.g. confidence intervals)
☐	☒	For null hypothesis testing, the test statistic (e.g. *F*, *t*, *r*) with confidence intervals, effect sizes, degrees of freedom and *P* value noted<br>*Give P values as exact values whenever suitable.*
☒	☐	For Bayesian analysis, information on the choice of priors and Markov chain Monte Carlo settings
☒	☐	For hierarchical and complex designs, identification of the appropriate level for tests and full reporting of outcomes
☒	☐	Estimates of effect sizes (e.g. Cohen's *d*, Pearson's *r*), indicating how they were calculated

Our web collection on statistics for biologists contains articles on many of the points above.

Software and code

Policy information about availability of computer code

Data collection	No software was used for data collection.
Data analysis	Seurat v4.3.0 (https://satijalab.org/seurat/articles/pbmc3k_tutorial.html) was used in scRNA-seq, Data Integration, Spatial Transcriptomics, scMultiomics and scATAC-seq module. CELLiD (https://immunesinglecell.org/cellpredictor) and CellTypist (https://www.celltypist.org/) was used in scRNA-seq, Data Integration and scMultiomics module. GSEA (https://www.gsea-msigdb.org/gsea/index.jsp) was used in scRNA-seq, Data Integration, Spatial Transcriptomics, scMultiomics and scATAC-seq module. LIANA (https://saezlab.github.io/liana/) and CellPhoneDB (https://github.com/Teichlab/cellphonedb) was used in scRNA-seq and Data Integration module. Harmony (https://github.com/immunogenomics/harmony), scVI (https://scvi-tools.org/) and fastMNN (https://rdrr.io/github/LTLA/batchelor/man/fastMNN.html) was used in Data Integration module. GraphST (https://github.com/JinmiaoChenLab/GraphST) was used in Spatial Transcriptomics module. MOFA+ (https://github.com/bioFAM/MOFA2) was used in scMultiomics module. Signac (https://stuartlab.org/signac/) and rGREAT (https://github.com/jokergoo/rGREAT) was used in scATAC-seq module.

For manuscripts utilizing custom algorithms or software that are central to the research but not yet described in published literature, software must be made available to editors and reviewers. We strongly encourage code deposition in a community repository (e.g. GitHub). See the Nature Portfolio guidelines for submitting code & software for further information.

Data

Policy information about availability of data

All manuscripts must include a data availability statement. This statement should provide the following information, where applicable:

- Accession codes, unique identifiers, or web links for publicly available datasets
- A description of any restrictions on data availability
- For clinical datasets or third party data, please ensure that the statement adheres to our policy

ezSingleCell consists of five modules, namely the single-cell RNA-Seq (scRNA-Seq) module, single-cell data integration (scIntegration) module, spatial transcriptomics (ST) module, single-cell multiomics (scMultiomics) module and single-cell ATAC-seq (scATAC-Seq) module.

The single cell RNA-seq module accepts 3 data formats namely gene counts (in text format), 10X cellranger output and seurat object. For gene counts, the test dataset of 2,700 Human peripheral blood mononuclear cells (from Seurat guided clustering tutorial dataset) was downloaded from google drive link (https://drive.google.com/file/d/1zoCaPKI3mV_BOJM4RPpiO3zW949Y2azq/view?usp=drive_link). For 10X cellranger output, the test dataset can be downloaded from 10X website (https://cf.10xgenomics.com/samples/cell/pbmc3k/pbmc3k_filtered_gene_bc_matrices.tar.gz) or google drive link (https://drive.google.com/file/d/156o3WvxJi6XZkoiOgqTScOA4QlU_cjFZ/view?usp=share_link). For seurat object, the test dataset can be downloaded from google drive link (https://drive.google.com/file/d/1xQzSbYWqXanPcCVdP7wtWJO33RwmG28V/view?usp=drive_link). For anndata object, the test dataset can be downloaded from google drive link (https://drive.google.com/file/d/1tebBIZi9i8s8a63xUXP2dxRLhuJ_E4dq/view?usp=drive_link).

For data integration module, the test gene expression dataset comprising of 500 cells from each batch was downloaded from 10X website using the following links : Batch 1 - https://support.10xgenomics.com/single-cell-gene-expression/datasets/2.1.0/pbmc8k and Batch 2 - https://support.10xgenomics.com/single cell vdj/datasets/2.2.0/vdj_v1_hs_pbmc_5gex or google drive link (https://drive.google.com/file/d/1SxhS1q2SgIDSwnu-4_9x4vTpriwI4Z6T/view?usp=drive_link). The metadata can also be found at the google drive link (https://drive.google.com/file/d/1Cp9bJ0_DFqh7ePr7LruDeBj3P8ojpQ-1/view?usp=drive_link). For Cellranger output, the data can be found at the google drive link : Batch 1 data (https://drive.google.com/file/d/11yc2MvzQAlSvk_BCSr9LqQjZ1lXuEwRX/view?usp=drive_link) and Batch 2 data (https://drive.google.com/file/d/17ShgTTMx8k8IpENqKDOiPH6Fx-ns4JVX/view?usp=drive_link).

The Spatial Transcriptomics module of ezSingleCell offers analysis for 10X Visium and Xenium data. For 10X Visium, the test dataset of Human Breast Cancer Block A comprising of 3798 spots was downloaded from https://www.10xgenomics.com/resources/datasets/human-breast-cancer-block-a-section-1-1-standard-1-1-0 and Mouse Brain Sagittal Anterior comprising of 2695 spots was downloaded from https://www.10xgenomics.com/resources/datasets/mouse-brain-serial-section-1-sagittal-anterior-1-standard-1-1-0. For Xenium dataset, the test dataset was downloaded from https://cf.10xgenomics.com/samples/xenium/1.0.2/Xenium_V1_FF_Mouse_Brain_Coronal_Subset_CTX_HP/Xenium_V1_FF_Mouse_Brain_Coronal_Subset_CTX_HP_outs.zip.

For scMultiomics, the test dataset of CITE-Seq Peripheral blood mononuclear cells (PBMCs) from a healthy donor comprising of 7865 cells was downloaded from https://support.10xgenomics.com/single-cell-gene-expression/datasets/3.0.0/pbmc_10k_protein_v3 and the 10X MultiOme Peripheral blood mononuclear cells (PBMCs) dataset from a healthy donor comprising of 3012 cells was downloaded from https://support.10xgenomics.com/single-cell-multiome-atac-gex/datasets/1.0.0/pbmc_unsorted_3k?.

For scATAC-seq, the test dataset of 500 Peripheral blood mononuclear cells from a healthy donor was downloaded from https://www.10xgenomics.com/resources/datasets/500-peripheral-blood-mononuclear-cells-pbm-cs-from-a-healthy-donor-next-gem-v-1-1-1-1-standard-2-0-0.

The reference datasets used for cell type annotation using CELLiD and CellTypist was downloaded from DISCO database (https://www.immunesinglecell.org/) and CellTypist website (https://www.celltypist.org/models) respectively.

The data used in this study has been deposited to zenodo (https://zenodo.org/records/10609310) and Github page (https://github.com/JinmiaoChenLab/ezSingleCell2). A summary of the datasets is also available in Supplementary Table 3 of the manuscript.

Research involving human participants, their data, or biological material

Policy information about studies with human participants or human data. See also policy information about sex, gender (identity/presentation), and sexual orientation and race, ethnicity and racism.

Reporting on sex and gender	N.A.
Reporting on race, ethnicity, or other socially relevant groupings	N.A.
Population characteristics	N.A.
Recruitment	N.A.
Ethics oversight	N.A.

Note that full information on the approval of the study protocol must also be provided in the manuscript.

Field-specific reporting

Life sciences study design

All studies must disclose on these points even when the disclosure is negative.

Sample size	We used publicly available data in all figures. We analyzed 1 test dataset for each of the five module available in ezSingleCell, namely single-cell RNA-Seq (scRNA-Seq) module, single-cell data integration (scIntegration) module, spatial transcriptomics (ST) module, single-cell multiomics (scMultiomics) module and single-cell ATAC-seq (scATAC-Seq) module. In scRNA-seq module, we used a test dataset of 2,700 Human peripheral blood mononuclear cells (from Seurat guided clustering tutorial dataset). In Data Integration module, the test gene expression dataset comprising of 500 cells from each batch was downloaded from 10X website. The Spatial Transcriptomics module of ezSingleCell offers analysis for 10X Visium (comprising of 3798 spots) and Xenium data. The test dataset for both these technologies were downloaded from 10X website. Similarly, the test dataset for CITE-Seq Peripheral blood mononuclear cells (PBMCs) from a healthy donor comprising of 7865 cells and 10X MultiOme Peripheral blood mononuclear cells (PBMCs) dataset from a healthy donor comprising of 3012 cells in scMultiomics was downloaded from 10X website. Lastly, the test dataset of 500 Peripheral blood mononuclear cells from a healthy donor in scATAC-seq module was also downloaded from 10X website.
Data exclusions	No data was excluded from this study.
Replication	Since our ezSingleCell manuscript describes an easy-to-use application for analyzing various single-cell and spatial omics data types, we don't require replicates for this study.
Randomization	None of the analysis performed by ezSingleCell does not requires Randomization.
Blinding	None of the analysis performed by ezSingleCell does not requires Blinding.

Reporting for specific materials, systems and methods

We require information from authors about some types of materials, experimental systems and methods used in many studies. Here, indicate whether each material, system or method listed is relevant to your study. If you are not sure if a list item applies to your research, read the appropriate section before selecting a response.

Materials & experimental systems

n/a	Involved in the study
☒	☐ Antibodies
☒	☐ Eukaryotic cell lines
☒	☐ Palaeontology and archaeology
☒	☐ Animals and other organisms
☒	☐ Clinical data
☒	☐ Dual use research of concern
☒	☐ Plants

Methods

n/a	Involved in the study
☒	☐ ChIP-seq
☒	☐ Flow cytometry
☒	☐ MRI-based neuroimaging

Plants

Seed stocks	Since our ezSingleCell manuscript describes an easy-to-use application for analyzing various single-cell and spatial omics data types, we don't require seed stocks.
Novel plant genotypes	Since our ezSingleCell manuscript describes an easy-to-use application for analyzing various single-cell and spatial omics data types, we don't require novel plant genotypes.
Authentication	Since our ezSingleCell manuscript describes an easy-to-use application for analyzing various single-cell and spatial omics data types, we don't require authentication.
